# Comparison of uniaxial and triaxial accelerometry in the assessment of physical activity among adolescents under free-living conditions: the HELENA study

**DOI:** 10.1186/1471-2288-12-26

**Published:** 2012-03-12

**Authors:** Jérémy Vanhelst, Laurent Béghin, Alain Duhamel, Patrick Bergman, Michael Sjöström, Frédéric Gottrand

**Affiliations:** 1 Inserm U995, IFR114, Faculty of medicine, University Lille 2, Lille, France; 2 CIC-9301-CHRU-INSERM, University hospital, Lille, France; 3 Department of Biostatistic, CHU Lille, Univ Lille Nord de France, F-59000 Lille, France; 4 Unit for Preventive Nutrition, Department of Biosciences and Nutrition, Karolinska Institute, Huddinge, Sweden; 5 School of ZEducation, psychology and sport sciences, Linneaus University, Kalmar, Sweden

**Keywords:** Accelerometers, Human locomotion, Energy expenditure, Youth

## Abstract

**Background:**

Different types of devices are available and the choice about which to use depends on various factors: cost, physical characteristics, performance, and the validity and intra- and interinstrument reliability. Given the large number of studies that have used uniaxial or triaxial devices, it is of interest to know whether the different devices give similar information about PA levels and patterns. The aim of this study was to compare physical activity (PA) levels and patterns obtained simultaneously by triaxial accelerometry and uniaxial accelerometry in adolescents in free-living conditions.

**Methods:**

Sixty-two participants, aged 13-16 years, were recruited in this ancillary study, which is a part of the Healthy Lifestyle in Europe by Nutrition in Adolescence (HELENA). All participants wore a uniaxial accelerometer (ActiGraph GT1M^®^, Pensacola, FL) and a triaxial accelerometer (RT3^®^, Stayhealthy, Monrovia, CA) simultaneously for 7 days. The patterns were calculated by converting accelerometer data output as a percentage of time spent at sedentary, light, moderate, and vigorous PA per day. Analysis of output data from the two accelerometers were assessed by two different tests: Equivalence Test and Bland & Altman method.

**Results:**

The concordance correlation coefficient between the data from the triaxial accelerometer and uniaxial accelerometer at each intensity level was superior to 0.95. The ANOVA test showed a significant difference for the first three lower intensities while no significant difference was found for vigorous intensity. The difference between data obtained with the triaxial accelerometer and the uniaxial monitor never exceeded 2.1% and decreased as PA level increased. The Bland & Altman method showed good agreement between data obtained between the both accelerometers (*p *< 0.05).

**Conclusions:**

Uniaxial and triaxial accelerometers do not differ in their measurement of PA in population studies, and either could be used in such studies.

## Background

Physical activity (PA) is essential in health promotion and disease prevention. PA protects against many diseases such as mental, nutritional, gastroenterological, cardiac, and respiratory diseases [1,2].

Daily PA may be measured by several methods. Accelerometry is a precise, reproducible, noninvasive, and relatively low-cost method that can be used with minimal interference in free-living conditions [3]. An accelerometer detects bodily acceleration, which is represented as an analog voltage created by a piezoelectric instrument that is sensitive to compression in a vertical direction. The signal is then summarized over a user-defined time, called an epoch, into what are called "counts" [4]. The higher the count, the higher is the intensity of PA. Accelerometers can assess the intensity, frequency, and duration of PA, and can thus be used to describe both the level (sum) and the pattern (distribution at various intensities over a defined period, such as a day or week) of PA.

Different types of devices are available and the choice about which to use depends on various factors: cost (especially when large populations are studied), physical characteristics (weight, size, and battery life), performance (number of axes, possible epochs, system of data transfer, recording duration, function of the epochs, and the memory capacity), and the validity and intra- and interinstrument reliability. Given the large number of studies that have used uniaxial or triaxial devices, it is of interest to know whether the different devices give similar information about PA levels and patterns; if so, this would allow for the comparison of outcomes and conclusions between studies. Indeed, the raw data of the accelerometry, generally expressed in counts, are very different depending on accelerometers used. The magnitude of the counts depends on the electrical and mechanical properties, which are different between RT3 and actigraph. Specially since the actigraph only measures acceleration (movement) in the vertical axis it could be hypothesized that it could miss some activities in children, even if the magnitude of difference between the 2 accelerometers cannot be a priori established from previously published studies.

The aim of this study was to compare PA levels and patterns assessed simultaneously by a triaxial accelerometer and a uniaxial accelerometer in adolescents in free-living conditions.

## Methods

This is an ancillary study that is part of the Healthy Lifestyle in Europe by Nutrition in Adolescence Cross-Sectional Study (HELENA-CSS) performed in European adolescents [5]. The present study comprises 62 (39 girls and 23 boys) healthy Caucasian adolescents aged 12.5-17.5 years from Lille (France) who provided complete accelerometer data. The mean ± SD for age, body mass, and stature were 14.2 ± 1.1 years, 60 ± 11 kg, and 168 ± 8 cm for boys, and 13.9 ± 0.9 years, 54 ± 8 kg, and 162 ± 7 cm for girls, respectively. Mean weight/age Z score was 1.1, Height/age Z score was 0.9 and Z score BMI was 0.7 [6]. One adolescent was obese and eight were overweighed. Puberty was assessed using Tanner staging: 3 were grade 2, 20 grade 3, 27 grade 4, and 12 have finished puberty [7]. The aims and objectives were explained carefully to each subject. Written, informed consent was obtained from the children and their parents. Participation in the study was voluntarily. The study was approved by the Ethics Committee of Lille (Comité de Protection des Personnes, Lille, France), and all procedures were performed in accordance with the ethical standards of the Helsinki Declaration of 1975 as revised in 2008 and the European Good Clinical Practices [8].

### Procedures

All participants wore the uniaxial accelerometer and the triaxial accelerometer simultaneously for 7 days. They were instructed to remove the devices during swimming, showering, and the bathing. The accelerometers recorded activity during the day, and were taken off at night. Mean duration of data record available per day for analysis was 11.5 ± 2.5 h.day^-1^. Each accelerometer was calibrated according to the manufacturer's recommendation according to each participant's body mass, stature, and age. Both accelerometers were attached to a belt and were worn on the right hip.

### Measurements

#### Triaxial accelerometer

The triaxial accelerometer used was the RT3^® ^accelerometer (Stayhealthy Inc., Monrovia, CA), which measures 71 × 56 × 28 mm and weighs 62.5 g [9]. It measures acceleration and deceleration in the three dimensions of space according to a vertical vector (*x*), an anteroposterior vector (*y*), and a mediolateral vector (*z*). The vector magnitude (VM) is calculated as the square root of the sum squared of activity counts in each vector. The epoch interval for the accelerometer was set at 1 min, and the output was expressed as mean counts of VM⋅min^-1^. Data were uploaded from the monitor to a computer after the fulfilled registration period (1 week). To measure PA patterns, we used recently established thresholds measured in an independent population of adolescents [10]. The PA categories and corresponding accelerometer data were: sedentary activity, 0-40 counts⋅min^-1^; light activity, 41-950 counts⋅min^-1^; moderate activity, 951-3,410 counts⋅min^-1^; and vigorous activity, > 3,410 counts⋅min^-1^. These thresholds have been validated against spiro-ergometry and heart rate monitoring [10]. The intra and inter-instrument reliability is low for activity of low magnitude and frequency, and better for moderate and vigorous activities [11-13]. The reliability of RT3 is however considered sufficient to assess PA [12].

#### Uniaxial accelerometer

The uniaxial accelerometer used was the ActiGraph^® ^Monitor (ActiGraph GT1M^®^, Pensecalo, FL), which measures 51 × 41 × 15 mm and weighs 43 g. The epoch interval for the ActiGraph monitor was also set at 1 min, and the output was expressed as counts⋅min^-1^. As for the RT3 accelerometer, data were uploaded from the monitor to a computer after 1 week. The PA categories and corresponding accelerometer data were: sedentary activity, 0-400 counts⋅min^-1^; light activity, 401-1,900 counts⋅min^-1^; moderate activity, 1,901-3,918 counts⋅min^-1^; and vigorous activity > 3,918 counts⋅min^-1^. These thresholds were validated against oxygen consumption and heart rate [14]. The reliability of this device is high for both sedentary activities than for vigorous activities [15].

### Statistical analysis

Quantitative variables were described by mean and 95% confidence interval [lower; upper].

For each intensity, reproducibility between RT3 accelerometer and uniaxial accelerometer was assessed with intraclass correlation coefficient (ICC) for continuous variables. The scale used for interpretation of this concordance was that described by Fleiss [16]. A concordance value greater than 0.8 was considered as good agreement. The reproductibility between the two accelerometers was also investigated on a diagram according to the method of Bland and Altman [17]. Then for each intensity, differences between values of RT3 accelerometer and uniaxial accelerometer were studied by a linear mixed model in order to take into account the correlation between the repeated measurements for a subject. Fixed effects were the day of measurement and the type of accelerometer, subject effect was considered as random.

Statistical analyses were performed using SAS software version 9.2 (SAS Institute Inc., Cary, NC 25513). *P *values < 0.05 were considered statistically significant.

## Results

The time registered by monitors was 900 min ⋅ day^-1^. Mean PA was 204.1 ± 104.3 counts⋅ h^-1 ^for the RT3^® ^and 353.2 ± 240 counts h^-1 ^for the ActiGraph^®^. Percentages of time spent at different PA levels are shown in Table 1. Both accelerometers showed that the adolescents spent about 50% of their time performing sedentary activities, 40% performing light PA, 9% moderate PA, and < 1% vigorous PA.

**Table 1:** Time spent (%) in various intensities assessed between triaxial and uniaxial accelerometer (n = 62)

Intensity	Monitor	Mean [95% IC]	Mean difference [95% IC]	ICC	P value
**Sedentary**	Triaxial	48.99 [47.32; 50.65]			

	Uniaxial	51.13 [49.46; 52.79]	2.06 [1.86; 2.26] ^†^	0.99	0.0187

**Light**	Triaxial	41.12 [39.76; 42.48]			

	Uniaxial	39.61 [38.26; 40.97]	- 1.43 [-1.62; -1.25] ^††^	0.99	0.0323

**Moderate**	Triaxial	9.53 [8.90; 10.17]			
	Uniaxial	8.84 [8.20; 9.47]	- 0.69 [-0.77; -0.61] **	0.99	0.0323

**Vigorous**	Triaxial	0.36 [0.29; 0.44]			

	Uniaxial	0.34 [0.27; 0.42]	- 0.02 [-0.04; 0.01] *	0.95	0.7108

The concordance correlation coefficient between the data from the triaxial accelerometer and uniaxial accelerometer at each intensity level was superior to 0.95 (Table 1). The ANOVA test showed a significant difference for the first three lower intensities while no significant difference was found for vigorous intensity (Table 1). The difference between data obtained with the triaxial accelerometer and the uniaxial monitor never exceeded 2.1% and decreased as PA level increased (Table 1).

**Figure 1:**
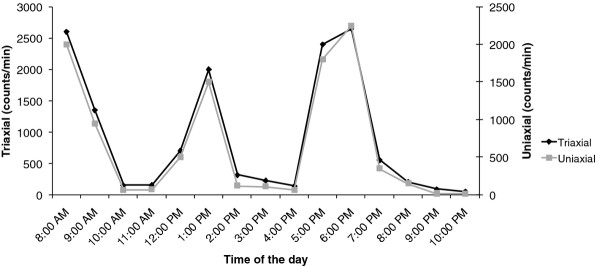
**Comparisons (averaged by hour) between Triaxial and Uniaxial in a representative subject on 1 day**.

**Figure 2:**
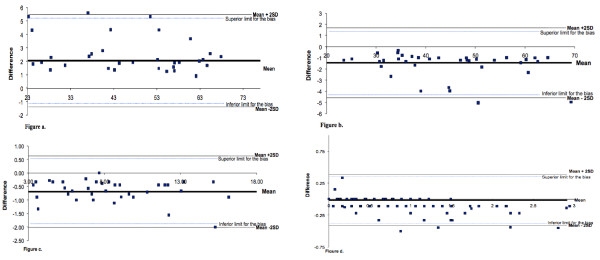
Difference of time (%) spent at different levels PA (a. Sedentary; b. Light; c. Moderate; d. Vigorous) assessed between triaxial and uniaxial accelerometer.

Figure 1 shows PA pattern of a representative subject during 1 day of week. Agreement at several levels was obtained because the mean difference was within the limits of agreement and most data points were within the limits of agreement of bias (Figure 2). Six participants were over to +2SD or under -2SD of the mean difference between the both accelerometer assessing the time spent at different PA levels. Only 6 participants were over +2SD or under -2SD of the mean difference between the two accelerometer (Figure 2). They did not differ from the rest of the population, being 13.2 ± 0.6 years, 62 ± 7 kg, and 163 ± 4.2 cm for age, body mass, and stature, respectively. Whether or not these outliers were engaged in specific sports activities could unfortunately not be assessed.

## Discussion

The present study demonstrates a good reliability of PA obtained by the triaxial accelerometer and the uniaxial accelerometer in adolescents in free-living conditions. Indeed, even if significant differences between the triaxial and uniaxial devices were found for sedentary, light and moderate activities, these differences were small (less than 2.1%) and decreased with PA intensity level. Such low difference appears to be negligible from a clinical research point of view. Moreover, the Intra Class Correlation that is a measure to express consistency and agreement between 2 methods was superior to 95%, demonstrating a very good reliability between uniaxial and triaxial accelerometers. Therefore, these data indicate that these two devices assess PA level in a similar way and that data obtained with these two devices in different studies on PA patterns could be considered comparable.

Results from different studies showed that the triaxial accelerometer was more precise than uniaxial accelerometer to assess PA and energy expenditure in children and adults [18,19]. We also hypothesized that the triaxial accelerometer could assess PA levels better because it measures movements in the three dimensions of space whereas the uniaxial accelerometer measures only in one dimension, and so it may lack some movements [20]. However, our results demonstrate that the uniaxial is as efficient as the triaxial accelerometer in the assessment of PA levels in FLC in adolescents. One hypothesis explaining this discrepancy could be the low impact of anteroposterior and mediolateral axis in the calculation of the VM using the triaxial accelerometer. This is supported by a recent study showing that the vertical axis (x) predicts similarly the activity energy expenditure than the VM (which is the square root of the sum squared of activity counts in each vector) [21]. Therefore, the vertical axis seems the most important one in the assessment of PA using the triaxial accelerometer, and compares the uniaxial accelerometer.

Our results agree with those of Macfarlane et al., who compared the validity of six methods to assess PA in daily life using questionnaires, heart rate monitoring, and accelerometers [22]. In the study by Macfarlane et al., the same accelerometers were worn for seven consecutive days by 49 Chinese subjects aged 15-55 years. As in the present study, they found that the triaxial accelerometer RT3 and the uniaxial accelerometer ActiGraph gave similar results for time spent in light, moderate, and vigorous activity. One important additional point in our study is that the differences between the two devices are also low (2.1%) for sedentary PA, which was the predominant level of PA for most of the adolescents; even a minimal difference in this category could yield a large difference in terms of total PA. Paul et al. showed also, although these two types of accelerometer (ActiGraph and Actical) predict PA on the same scale (counts ⋅min^-1^), the results differed [23]. This agrees with our findings because total PA was 204.136 counts ⋅ 24 h^-1 ^for the RT3 and 353.252 counts ⋅ 24 h^-1 ^for the ActiGraph. However, our results show clearly that these two devices distinguish different intensities of PA in the same manner.

Two studies have been previously performed in adults to compare different prediction equations of energy expenditure using both RT3 and Actigraph [24,25]. The first study was performed in adults on the capabilities of eight previously published regression equations for three commercially available accelerometers (ActiGraph, Actical and RT3) to predict daily energy expenditure [24]. Eighty-five subjects completed one overnight stay in a room calorimeter where they engaged in typical activities in free living conditions (walking, jogging, deskwork...). The authors concluded that most energy expenditure prediction equations showed differences of < 2% in the moderate and vigorous intensity categories, while, several regression equations under or overstimated the energy expenditure against the direct calorimetry [24]. The second study was realized in 13 subjects during 7 days where the total daily energy expenditure was measured with ActiGraph and Tritrac (predecessor of the RT3 accelerometer) regression equations against the doubly labeled water [25]. Of the 14 different regression equations examined, only two developed for the ActiGraph accelerometer were not significantly different from the doubly labeled water method. All equations for the RT3 accelerometer showed significant difference with the doubly labeled water method. Authors concluded that the results from these two studies imply that researchers may want to avoid using accelerometers to predict energy expenditure in free-living conditions, instead using these instruments only to measure patterns of PA. Our results reinforce this conclusion showing good concordance and agreement of the two accelerometers in the assessment of PA patterns.

A 2 or 5 s epoch is preferred for assessing PA in children since most of their spontaneous activities are very short and do not exceed one minute [26]. A 15 s epoch can be used with adolescents [27]. However due to technical constraints (RT3 does not have the capacity to record data for 1 week with a short second epoch) a 1 min epoch was chosen for the study. Whether or not using a 1 s epoch would change the comparison between these 2 devices is unknown but we could speculate it would not change significantly the comparison of the devices but rather the physical activity patterns.

In the present study, we used predictive equations obtained previously in our group for the two accelerometers. However, other groups have published different predictive equations, we cannot exclude that the results of the comparison between these two accelerometers should have been different using other equations [27-29].

## Conclusions

In summary, this study demonstrates a strong reliability between PA levels and patterns obtained by a triaxial accelerometer (RT3^®^) and a uniaxial accelerometer (ActiGraph^®^) in adolescents in free-living conditions. The choice of a uniaxial or triaxial accelerometer makes little difference in the assessment of PA pattern in free-living conditions. Therefore, both uniaxial or triaxial accelerometers can be used in clinical practice to quantify PA.

## Competing interests

The authors declare that they have no competing interests.

## Authors' contributions

LB, FG, JV conceived the idea. JV, LB and FG wrote the first and subsequent drafts. AD, PB and MS helped developed the ideas. All authors read and approved the final manuscript.

## Pre-publication history

The pre-publication history for this paper can be accessed here:

http://www.biomedcentral.com/1471-2288/12/26/prepub
